# The Swedish version of the Anterior Cruciate Ligament Quality Of Life measure (ACL-QOL): translation and measurement properties

**DOI:** 10.1007/s11136-022-03265-1

**Published:** 2022-10-13

**Authors:** Stephanie R. Filbay, Hanna Tigerstrand Grevnerts, Sofi Sonesson, Henrik Hedevik, Joanna Kvist

**Affiliations:** 1 Centre for Health, Exercise and Sports Medicine, Department of Physiotherapy, University of Melbourne, Melbourne, Australia; 2 Unit of Physiotherapy, Department of Health, Medicine and Caring Sciences, Linköping University, Linköping, Sweden; 3 Stockholm Sports Trauma Research Center, Dept of Molecular Medicine & Surgery, Karolinska Institutet, Solna, Sweden

**Keywords:** Anterior cruciate ligament reconstruction, Non-operative management, Validity, Reliability, Responsiveness, Measurement properties

## Abstract

**Purpose:**

To translate the ACL-QOL from English to Swedish and evaluate measurement properties for use after surgical and non-surgical management of anterior cruciate ligament (ACL) injury.

**Methods:**

The ACL-QOL was translated from English to Swedish and data were pooled from 13 cohorts to enable a comprehensive evaluation of measurement properties in line with COSMIN guidelines. We evaluated internal consistency, test–re-test reliability, measurement error, structural validity [confirmatory factor analysis (CFA)], construct validity and responsiveness (hypothesis testing), and floor/ceiling effects. Results were stratified by time since injury (≤ 1.5 years; 2–10 years, 15–25 years; > 30 years) and ACL management strategy [surgical (*n* = 1163), non-surgical (*n* = 570)].

**Results:**

The Swedish ACL-QOL had sufficient internal consistency (total and domain scores) for use in surgically managed (Cronbach’s alpha ≥ 0.744) and non-surgically managed (≥ 0.770) ACL-injured individuals at all time-points. Test–re-test reliability was sufficient [intraclass correlation coefficients: all domains > 0.80, total score 0.93 (95% CI 0.86–0.96)]. The standard error of measurement was 5.6 for the total score and ranged from 7.0 to 10.3 for each domain. CFA indicated sufficient SRMR values when using the total score or five domains; however, CFI and RMSEA values did not meet cut-offs for good model fit. Hypothesis testing indicated sufficient construct validity and responsiveness. Floor effects were negligible and ceiling effects were negligible or minor.

**Conclusion:**

The Swedish version of the ACL-QOL has sufficient internal consistency, test–re-test reliability, construct validity and responsiveness, for use in people with ACL injury managed with or without ACL surgery. Model fit could be improved and investigation into the source of misfit is warranted.

**Supplementary Information:**

The online version contains supplementary material available at 10.1007/s11136-022-03265-1.

## Introduction

Following an anterior cruciate ligament (ACL) rupture, many individuals experience long-term quality of life (QOL) impairment, irrespective of surgical or non-surgical management [1, 2]. The primary objectives of ACL injury management are to restore knee function, facilitate return to physical activity participation, prevent further injury and osteoarthritis, and optimise long-term quality of life [3]. This highlights the importance of a valid and reliable measure of knee-related QOL for use in ACL-injured individuals. The ACL-QOL is the only knee-related QOL measure designed specifically for use in ACL-injured individuals [4], and contains more items of relevance to ACL-injured individuals than other commonly used knee measures [5]. The ACL-QOL comprises 32 items across 5 domains (symptoms and physical complaints, work-related concerns, sport and recreational concerns, lifestyle issues, and social and emotional concerns). Each item is scored on a visual analogue scale from 0 to 100, and a score for each domain as well as an overall score in the range of 0 (worst possible score) to 100 (best possible score) is calculated where each item is weighted equally. Although the ACL-QOL was initially designed and validated for use in people with chronic ACL deficiency, it has become a common measure of knee-related QOL for use in ACL-reconstructed patients with further testing of measurement properties within this population [6].

There is a high incidence of ACL injury in Sweden [7], and a valid and reliable measure of knee-related QOL for use in ACL-injured people who speak Swedish as a first language is needed. Although a Swedish version of the ACL-QOL has been used in previous research [8–20], the translation and development procedure of the Swedish version of the ACL-QOL has not been published and measurement properties have not been evaluated. Assessing the methodological quality of a measurement instrument is required to help clinicians and researchers determine whether it is appropriate for use in specific populations and whether the results are trustworthy [21].

The aim of this study was to translate the ACL-QOL from English into Swedish and perform a comprehensive evaluation of the measurement properties of the Swedish version of the ACL-QOL. To determine the appropriateness for use of the Swedish ACL-QOL, we evaluated measurement properties for use in both surgically and non-surgically managed individuals with ACL injury, at multiple time points post-injury (ranging from the acute period to 37 years after ACL injury).

## Materials and methods

### The construct

The ACL-QOL was developed to measure knee-specific health-related QOL. Health-related QOL has been defined as “the impact of disease and treatment on disability and daily functioning”, reflecting “the impact of perceived health on an individual’s ability to live a fulfilling life” [22]. Thus, the ACL-QOL should evaluate ‘the impact of the ACL-injured knee on an individual’s ability to live a fulfilling life.’

### Translation procedure

The translation of the ACL-QOL was completed in accordance with guidelines recommended to preserve equivalence in cross-cultural adaptation of health measures [23]. First, permission was gained by the instrument developer, Nick Mohtadi (University of Calgary Sport Medicine Centre, Calgary, Alberta, Canada), to translate and culturally adapt the ACL-QOL from English to Swedish. The English version of the ACL-QOL was translated to Swedish by a physiotherapist with experience managing ACL-injured individuals, who was a native Swedish speaker fluent in English. Adjustments were made following discussion and review of each item with the senior author (JK) who is a physiotherapist with research and clinical expertise in the management of ACL injury. The English and Swedish versions of the ACL-QOL were then sent to a bilingual multidisciplinary committee with expertise researching and/or treating ACL-injured individuals. The committee consisted of a senior orthopaedic surgeon, three physiotherapists, two senior physiotherapist researchers, and a professor of physiotherapy at the University of Linköping, Sweden.

Following this, a committee meeting was held with the multidisciplinary group where the translation and relevance of content (i.e. the content validity) was discussed in depth. This meeting resulted in several small adaptations to wording to ensure items were culturally appropriate for Swedish respondents whilst maintaining their original meaning. Specific focus was placed on ensuring the translated version was written in clear and comprehensible Swedish. Discussion at the committee meeting generated the addition of one new item ‘How limited is your sex life because of your knee?’ Clinicians who participated in the meeting recognised this as a common concern raised by ACL-injured patients with potential to negatively impact QOL, and this had not been addressed in the English version of the ACL-QOL.

Following this, the Swedish version of the ACL-QOL was tested on three individuals with non-surgically treated ACL injury, within 12 months of ACL injury. The ACL-QOL was completed with a physiotherapist researcher present and the respondents were encouraged to discuss their understanding and interpretation of the questions, raise any issues with the questionnaire and discuss the relevance of the content. This process did not result in any changes to the questionnaire since respondents did not raise any concerns. The Swedish version of the ACL-QOL was then re-sent to the multidisciplinary committee. After further minor grammatical adjustments the ACL-QOL was back-translated from Swedish to English by a certified translator who was a native English speaker fluent in Swedish. The two English versions and the Swedish version were compared by two physiotherapist researchers and no further adjustments were made.

### The Swedish version of the ACL-QOL

The original Swedish version of the ACL-QOL contained an additional item (‘How limited is your sex life because of your knee?’), resulting in 33 items instead of 32. The Swedish Version of the ACL-QOL (with and without the additional item) is provided in Online Resource 1. Since the measurement properties of the 33-item version of the Swedish ACL-QOL had not been investigated previously, we performed all analyses with and without this additional item. We found that the original 32-item version had better internal consistency of the social/emotional domain, similar test–retest reliability, measurement error, structural validity, construct validity, responsiveness, and floor/ceiling effects. In this manuscript, we provide results for the 32-item version of the Swedish ACL-QOL, and the results of measurement properties assessment for the 33-item version are presented as an Online Resource (See Online Resource 2). Each ACL-QOL item is scored on a visual analogue scale (VAS) from 0 (worst possible score) to 100 (best possible score). Items are evenly weighted, and a total score (or subscale score) is calculated by dividing the total score of all items by the total number of items. For convenience when using digital questionnaires, this can be converted to a 10-point scale [ranging from 1 (worst possible score) to 10 (best possible score)].

### Participants

We pooled data from 13 different cohorts (including 2 randomised controlled trials [24, 25], 6 prospective cohort studies [8–15, 26, 27] and 7 cross-sectional studies[16–20, 28]) to enable a comprehensive evaluation of ACL-QOL measurement properties for use in surgically and non-surgically managed ACL-injured individuals at varying timepoints following injury. Analyses of measurement properties for the ACL-QOL was approved by the Regional Ethical Review Board DNR: 21-04. All participants provided written informed consent for use of their anonymized data for research purposes. Additional information on each cohort including ethical approval, is presented in Online Resource 3. The rationale for investigating measurement properties separately for surgically and non-surgically managed individuals was the potential for different impacts on knee-related QOL following each management strategy. Additionally, the ACL-QOL is frequently used following ACLR despite its intended use in ACL deficient (non-surgically managed) individuals. Investigating measurement properties stratified by management strategy will inform future use of this instrument in research and clinical settings.

In total, 1733 questionnaires were analysed. Results are stratified by ACL management (surgical management *n* = 1163, non-surgical management *n* = 570), and by time since ACL injury or ACL surgery ≤ 1.5 years (surgical management *n* = 598, non-surgical management *n* = 339); 2–10 years (surgical management *n* = 370, non-surgical management *n* = 121); 15–25 years (surgical management *n* = 42, non-surgical management *n* = 35); > 30 years follow-up (surgical management *n* = 112, non-surgical management *n* = 66) (Table 1). Data from an additional 50 individuals that were outside of these time-intervals (surgical management *n* = 41, non-surgical management *n* = 9), were included in the confirmatory factor analysis and hypothesis testing. Participants ranged from 4 weeks post ACL injury to 37 years after ACL injury. Although some participants answered questionnaires at multiple time-points, only one questionnaire per participant was included in analyses (except for test–retest reliability and responsiveness where longitudinal data was used).

**Table 1:** Participant characteristics stratified by time since ACL injury (non-surgical) or time since ACL surgery

	** ≤ **1.5 year follow-up (*n* = 937)		2–10 year follow-up (*n* = 491)		15–25 year follow-up (*n* = 77)		> 30 year follow-up (*n* = 178)		All participants (*n* = 1,683)	
	Surgical (*n* = 598)	Non-surgical (*n* = 339)	Surgical (*n* = 370)	Non-surgical (*n* = 121)	Surgical (*n* = 42)	Non-surgical (*n* = 35)	Surgical (*n* = 112)	Non-surgical (*n* = 66)	Surgical (*n* = 1122)	Non-surgical (*n* = 561)
Sex, female	384 (64%)^0^	173 (51%)^2^	197 (53%)^0^	56 (46%)^0^	12 (29%)^0^	12 (34%)^0^	29 (26%)^0^	19 (29%)^0^	622 (55%)^0^	260 (47%)^2^
Age at follow-up, years	24 (7)^2^	27 (8)^5^	27 (8)^1^	31 (9)^0^	45 (5)^0^	43 (4)^0^	59 (6)^0^	59 (7)^0^	29 (13)^3^	33 (13)^5^
Symptoms/physical domain	81 (16)^4^	70 (19)^0^	78 (18)^4^	76 (20)^0^	82 (19)^1^	81 (18)^1^	78 (20)^1^	76 (20)^0^	80 (17)^10^	72 (20)^1^
Work-related domain	69 (23)^10^	58 (26)^3^	72 (23)^2^	70 (25)^0^	80 (20)^0^	81 (21)^0^	74 (23)^0^	77 (22)^0^	71 (23)^12^	64 (26)^3^
Recreational/sports domain	56 (25)^8^	39 (23)^5^	59 (24)^2^	50 (25)^1^	67 (23)^0^	60 (31)^1^	59 (28)^0^	60 (27)^0^	58 (25)^10^	45 (26)^7^
Lifestyle domain	65 (23)^11^	51 (24)^6^	69 (23)^3^	66 (24)^0^	79 (20)^0^	73 (22)^0^	68 (26)^1^	70 (25)^1^	67 (24)^15^	58 (25)^7^
Social/emotional domain	54 (23)^11^	46 (23)^7^	61 (25)^3^	59 (24)^1^	81 (19)^0^	74 (25)^0^	72 (25)^0^	73 (24)^0^	59 (25)^14^	54 (26)^8^
ACL-QOL total score	63 (20)^15^	50 (20)^6^	66 (21)^5^	61 (22)^0^	76 (18)^0^	70 (24)^0^	68 (23)^0^	69 (22)^0^	65 (21)^20^	56 (22)^6^

Descriptives are count (%) or mean (standard deviation); numbers in superscript represent the number of cases with missing data

### Outcome measures

The ACL Return to Sports After Injury (ACL-RSI) [29], International Knee Documentation Committee Subjective Knee Form (IKDC) [30], Knee Injury and Osteoarthritis Outcome Score (KOOS) Pain and Sport/Rec domains [31], Optum SF™ Health Surveys SF-36 (SF36) Physical Component Score (PCS) and Mental Component Score (MCS) [32], the EuroQol-5D (EQ-5D) [33], satisfaction with knee function, return to pre-injury sport, and perceived global rating of change were used for hypothesis testing and evaluating responsiveness.

The ACL-RSI measures ACL-injured individuals’ emotions, confidence, and risk appraisal in relation to return to sport, and scores range from 0 to100 (100 = no negative psychological response) [29]. The Swedish version of the ACL-RSI was found to have good face validity, internal consistency, high construct validity, low floor and ceiling effects and high reproducibility, for use in ACL-injured individuals [18]. The IKDC is intended to measure knee symptoms, knee function, and sports activities due to knee impairment [30]. Scores range from 0 to 100 (100 = no symptoms or limitations with activities). Both the original and the Swedish version of the IKDC have adequate internal consistency, test–re-test reliability and construct validity, for use in people with ACL injury [19, 34]. The KOOS was originally developed in Swedish, and intended for use in young, middle-aged, and elderly adults with knee injury and/or knee osteoarthritis, and comprises 5 subscales, with scores ranging from 0 to 100 (best possible score) [31]. The KOOS Pain and Sport/Recreational Function (Sport/Rec) subscale were used in analyses, these subscales have adequate internal consistency and test–retest reliability for use in ACL-injured cohorts [35].

The SF36 is a generic measure of health-related QOL, that assesses eight domains, enabling the calculation of two summary scores: PCS (physical functioning, bodily pain, general health perceptions and physical role limitation), and MCS (vitality, emotional role functioning, social role functioning, and mental health) [32]. The EQ-5D is a generic health-related QOL measure, comprising five items evaluating mobility, self-care, usual activities, pain/discomfort, and anxiety/depression [33]. Items are summed to provide an overall weighted ‘health-status’ utility score, where 0 represents ‘death’ and 100 represents ‘perfect health’ [33]. Satisfaction with knee function was measured with the question: ‘If you were to spend the rest of your life with your knee function just the way it has been in the last week, would you feel’ (response options were on a 7-point ordinal scale ranging from ‘delighted’ to ‘terrible’). Return to pre-injury sport was evaluated by a direct question to ACL-injured individuals [‘Have you returned to the same physical activity as before your injury?’ (yes/no)]. Patients’ perceived global rating of change was measured on a 6-point ordinal scale ranging from ‘fully recovered’ to ‘great deterioration’ [36].

### Measurement properties

Measurement properties for the Swedish version of the ACL-QOL were evaluated and reported in line with the COnsensus-based Standards for the selection of health Measurement INstruments (COSMIN) Study Design checklist [37]. Measurement properties were interpreted in line with the COSMIN updated criteria for good measurement properties [38]. For assessment of measurement properties (with the exception of factor analysis), a sample size of 100 is considered very good [37]. For assessment of structural validity using a factor analysis, a sample size of 7 cases per item is recommended (i.e. ≥ *n* = 224 for the ACL-QOL) [37].

### Internal consistency

Internal consistency refers to the interrelatedness amongst items of the ACL-QOL that are expected to be interchangeable and highly correlated [39]. We assessed the internal consistency of all items and within each domain. A Cronbach’s alpha statistic is calculated for the overall score and for each domain separately, where a value ≥ 0.70 is interpreted as sufficient internal consistency, assuming there is evidence for sufficient structural validity. A Cronbach’s alpha > 0.90 indicates potential item redundancy [21].

### Test–re-test reliability and measurement error

Individuals who had an ACL injury or ACL surgery 3 months to 27 years previously, completed the ACL-QOL, on two occasions. The first questionnaire was completed during a visit to a physiotherapy department either for rehabilitation or research follow-up reasons. The second questionnaire was sent to the patient 1 week after the visit and was completed within 4 weeks. This time interval was chosen to minimise recall bias whilst reducing the likelihood for change in knee-related QOL. 41 out of 47 participants completed the questionnaire on both occasions (response rate 87%). Participants were aged a mean 31 (95% CI 28–35) years and 44% were female.

The 2-way random effect model (absolute agreement definition), single measure intraclass correlation coefficient (ICC 2,1) [40], was used for analysis of relative reliability. An ICC value ≥ 0.70 is considered sufficient reliability [37]. In order to describe absolute reliability, the standard error of measurement (SEM) was calculated (S_diff_/√2) [37, 41]. Measurement error represents the systematic and random error of a respondent’s score that is not attributed to true changes in the construct to be measured [39].

### Content validity

Content validity was addressed during the original development of the English version of the ACL-QOL by interviewing patients with chronic ACL deficiency, studying the relevant literature and including direct patient input throughout the instrument’s development (including item generation, item reduction, and pretesting stages) to ensure items were appropriate and relevant to ACL-injured individuals [4]. Additionally, an expert multidisciplinary panel with experience treating ACL-injured individuals contributed to the development of the ACL-QOL [4].

Content validity of the Swedish version of the ACL-QOL was addressed by involving ACL-injured individuals and a multidisciplinary committee with expertise in researching and managing ACL-injured individuals, throughout the translation and adaption process. This was done to ensure the Swedish version of the ACL-QOL comprehensively addressed aspects of knee-related QOL that were relevant to Swedish ACL-injured individuals.

### Structural validity

Structural validity refers to the degree to which ACL-QOL scores are an adequate reflection of the dimensionality of the construct to be measured [39]. Structural validity was assessed using a confirmatory factor analysis [maximum likelihood ‘ML’ estimator with full information maximum likelihood ‘FIML’ to handle missing data, using R (R Core Team, 2021)]. Fit-parameters assessed if the proposed model [i.e. 1 factor (the total ACL-QOL score) or 5 factor (the 5 domain scores)] was better than alternative models, using the comparative fit index (CFI), the root mean square error of approximation (RMSEA) and the standardized root mean square residual (SRMR). Since confirmatory or exploratory factor analysis has not been performed for the ACL-QOL[6], 8 competing CFA models were tested based on putative dimensions of the measure to determine whether it is appropriate to report scores for the 5 pre-specified domains, in addition to the total ACL-QOL score. A CFI close to 0.95 or higher, RMSEA close to 0.06 or lower, and a SRMR close to 0.08 or lower, are representative of good fitting models [42]. The confirmatory factor analysis was performed in surgically treated (*n* = 1163) and non-surgically treated (*n* = 570) ACL-injured cohorts.

### Hypothesis testing for construct validity

Hypothesis testing for construct validity is recommended when there is no ‘gold standard’ available for comparison [21]. Since the ACL-QOL is the only ACL specific, knee-related QOL measure there is no gold standard available. Construct validity refers to the degree to which ACL-QOL scores are consistent with hypotheses based on the assumption that the ACL-QOL validly measures knee-related QOL in ACL-injured individuals. Seven pre-defined hypotheses were assessed in non-surgically treated and surgically treated cohorts. Pre-defined hypotheses required at least a moderate positive correlation (*r* ≥ 0.30) between the ACL-QOL and SF-36 MCS, SF-36 PCS, KOOS-Pain, KOOS-Sport/Rec and EQ-5D scores. Additionally, at least a 10-point difference on the ACL-QOL was required between people who were ‘satisfied’ (delighted to mostly satisfied) vs ‘dissatisfied’ (mostly dissatisfied to terrible) with their knee function, and those who returned to pre-injury sport vs. those who did not. Confirmation of at least 75% of the predefined hypotheses was considered necessary to represent good construct validity [21]. The sample size for each hypothesis ranged from *n* = 131 to *n* = 1103 (Table 5).

### Responsiveness

Responsiveness refers to the ability of the ACL-QOL to detect change over time in ACL-specific knee-related QOL [39]. Responsiveness was assessed by testing of pre-defined hypotheses concerning mean differences or expected correlations between changes in ACL-QOL scores and changes in scores on other measures known to be responsive. We formulated 11 hypotheses for testing in surgically and/or non-surgically managed cohorts to evaluate responsiveness (Table 6). At least 75% of our predefined hypotheses should be met, in order to represent adequate responsiveness [38, 43].

### Floor and ceiling effects

The proportion of respondents scoring the lowest and highest possible score on the ACL-QOL, and for each ACL-QOL domain, was evaluated. Floor and ceiling effects have been classified as significant if ≥ 15%, moderate if 10% to < 15%, minor if 5% to < 10%, and negligible if < 5% of participants score the lowest or highest possible score on a measure [44].

### Missing data

In order for a specific ACL-QOL domain score to be included in the analysis, at least 33% of items within a given ACL-QOL domain had to have complete data. Additionally, for a total ACL-QOL score to be calculated, at least 4 of the 5 domains had to have ≥ 33% complete data. Data was managed in this way to allow for a total ACL-QOL score to be calculated for participants for whom the work-related domain was not applicable [*n* = 106 (6%) were not working for reasons unrelated to their knee] There was 1.5% (*n* = 26) missing data for the total ACL-QOL score, and missing data for each domain ranged from 0.6% (*n* = 11, symptoms/physical domain) to 1.4% (*n* = 23, social/emotional domain).

## Results

### Internal consistency

Table 2 demonstrates sufficient internal consistency for the Swedish ACL-QOL (total score and individual domain scores) for use in surgically managed (lowest Cronbach’s alpha, 0.744) and non-surgically managed (lowest Cronbach’s alpha, 0.770), ACL-injured individuals at all time-points.

**Table 2:** Internal consistency for the Swedish ACL-QOL

	All patients	Years since acl injury			
		≤ 1.5 years	2–10 years	15–25 years	> 30 years
**Surgical**					
Total ACL-QOL	0.971 (*n* = 1057)	0.970 (*n* = 542)	0.970 (*n* = 340)	0.963 (*n* = 41)	0.980 (*n* = 96)
Symptoms/physical	0.846 (*n* = 1153)	0.835 (*n* = 594)	0.844 (*n* = 366)	0.796 (*n* = 41)	0.880 (*n* = 111)
Work-related	0.759 (*n* = 1073)	0.744 (*n* = 548)	0.772 (*n* = 346)	0.777 (*n* = 42)	0.803 (*n* = 98)
Recreational/sports	0.954 (*n* = 1153)	0.953 (*n* = 590)	0.953 (*n* = 368)	0.933 (*n* = 42)	0.968 (*n* = 112)
Lifestyle	0.912 (*n* = 1148)	0.906 (*n* = 587)	0.910 (*n* = 367)	0.898 (*n* = 42)	0.944 (*n* = 111)
Social/emotional	0.898 (*n* = 1148)	0.877 (*n* = 587)	0.900 (*n* = 367)	0.868 (*n* = 42)	0.921 (*n* = 112)
**Non-surgical**					
Total ACL-QOL	0.975 (*n* = 528)	0.971 (*n* = 322)	0.972 (*n* = 112)	0.973 (*n* = 30)	0.977 (*n* = 57)
Symptoms/physical	0.838 (*n* = 569)	0.829 (*n* = 339)	0.848 (*n* = 121)	0.850 (*n* = 34)	0.838 (*n* = 66)
Work-related	0.819 (*n* = 539)	0.816 (*n* = 328)	0.781 (*n* = 114)	0.770 (*n* = 32)	0.763 (*n* = 58)
Recreational/sports	0.961 (n = 563)	0.954 (*n* = 334)	0.952 (*n* = 120)	0.973 (*n* = 34)	0.966 (*n* = 66)
Lifestyle	0.925 (*n* = 563)	0.911 (*n* = 333)	0.917 (*n* = 121)	0.911 (*n* = 35)	0.945 (*n* = 65)
Social/emotional	0.914 (*n* = 562)	0.896 (*n* = 332)	0.892 (*n* = 120)	0.898 (*n* = 35)	0.913 (*n* = 66)

Data represents the Cronbach’s alpha

### Test–re-test reliability and measurement error

Test–re-test reliability and measurement error are reported in Table 3. Intraclass correlation coefficients exceeded 0.70 for all domains and the total ACL-QOL score, indicating sufficient test–re-test reliability. The standard error of measurement was 5.6 for the total ACL-QOL score, and ranged from 7.0 to 10.3 for the ACL-QOL domains.

**Table 3:** Measurement error and test–re-test reliability of the ACL-QOL administrated on two occasions, 1–4 weeks apart

	*n*	Mean difference (95% CI)	SEM	ICC (95% CI)
ACL-QOL total score	36	− 4.2 (− 6.9 to − 1.5)	5.6	0.93 (0.86–0.96)
Symptoms/physical	41	− 3.4 (− 6.6 to − 0.2)	7.1	0.82 (0.68–0.90)
Work-related	37	3.4 (− 1.5 to 8.2)	10.3	0.81 (0.66–0.90)
Recreational/sports	40	− 4.5 (− 7.7 to − 1.3)	7.1	0.93 (0.87–0.96)
Lifestyle	40	− 5.6 (− 8.8 to − 2.4)	7.0	0.93 (0.87–0.96)
Social/emotional	37	− 6.8 (− 11.5 to − 2.1)	10.1	0.84 (0.71–0.92)

*SEM*   standard error of measurement, *ICC*  intraclass correlation coefficient

### Structural validity

The 1-factor and 5-factor model had sufficient SRMR values, for use in both surgically and non-surgically managed individuals (Table 4). However, CFI and RMSEA values did not meet the threshold for sufficient structural validity, suggesting that model fit could be improved and further investigation into the source of misfit is warranted. The 5 factor model performed better than the 1 factor model, supporting use of the 5 domain scores. Standardised factor loadings are reported in Online Resource 4.

**Table 4:** Fit indices for confirmatory factor analysis model

	CFI	RMSEA (95% CI)	SRMR	Chi-square (df), p value
Surgical (*n* = 1163)				
Model 1: 1 factor	0.790	0.110 (0.108–0.112)	0.063	6983.6 (464), < 0.001
Model 2A: 2 factor (symptoms + work, sport + lifestyle + SOCEMO)	0.826	0.102 (0.099–0.104)	0.057	6014.0 (463), < 0.001
Model 2B: 2 factor (symptoms + work + sport, lifestyle + socemo)	0.806	0.106 (0.103–0.108)	0.063	6482.1 (463), < 0.001
Model 3A: 3 factor (symptoms + work + sport, lifestyle, socemo)	0.821	0.102 (0.099–0.104)	0.061	6010.2 (461), < 0.001
Model 3B: 3 factor (symptoms + work, sport, lifestyle + socemo)	0.840	0.096 (0.094–0.098)	0.056	5406.7 (461), < 0.001
Model 4A: 4 factor (symptoms + work, sport, lifestyle, socemo)	0.856	0.091 (0.089–0.094)	0.054	4912.0 (458), < 0.001
Model 4B: 4 factor (symptoms, work, sport, lifestyle + socemo)	0.843	0.095 (0.093–0.098)	0.055	5312.4 (458), < 0.001
Model 5: 5 factor (symptoms, work, sport, lifestyle, socemo)	0.859	0.091 (0.089–0.093)	0.052	4818.2 (454), < 0.001
Non-surgical (*n* = 570)				
Model 1: 1 factor	0.838	0.100 (0.097–0.104)	0.056	3131.1 (464), < 0.001
Model 2A: 2 factor (symptoms + work, sport + lifestyle + socemo)	0.868	0.091 (0.087–0.094)	0.047	2641.0 (463), < 0.001
Model 2B: 2 factor (symptoms + work + sport, lifestyle + socemo)	0.852	0.096 (0.093–0.099)	0.056	2898.4 (463), < 0.001
Model 3A: 3 factor (symptoms + work + sport, lifestyle, socemo)	0.858	0.095 (0.091–0.098)	0.055	2808.0 (461), < 0.001
Model 3B: 3 factor (symptoms + work, sport, lifestyle + socemo)	0.890	0.083 (0.080–0.087)	0.045	2280.9 (461), < 0.001
Model 4A: 4 factor (symptoms + work, sport, lifestyle, socemo)	0.897	0.081 (0.077–0.084)	0.043	2161.2 (458), < 0.001
Model 4B: 4 factor (symptoms, work, sport, lifestyle + socemo)	0.895	0.081 (0.078–0.085)	0.044	2191.7 (458), < 0.001
Model 5: 5 factor (symptoms, work, sport, lifestyle, socemo)	0.902	0.079 (0.076–0.082)	0.042	2066.7 (454), < 0.001

*CFI*  comparative fit index, *RMSEA*   root mean square error of approximation, *SRMR*   standardized root mean square residual, *df*   degrees of freedom

### Hypothesis testing for construct validity

All predefined hypotheses were met for surgically and non-surgically managed groups (Table 5) representing sufficient construct validity.

**Table 5:** Hypothesis testing to evaluate construct validity

	Surgical		Non-surgical	
Hypothesis	*n* = *r/*MD (95% CI)		*n* = *r/*MD (95% CI)	
The ACL-QOL score should be at least moderately (*r* ≥ 0.30), positively correlated with the PCS SF-36 score	337	*r* = 0.70* 0.63–0.76	131	*r* = 0.70* 0.63–0.77
The ACL-QOL score should be at least moderately (r ≥ 0.30), positively correlated with the SF-36 MCS score	337	*r* = 0.39* 0.30–0.48	131	*r* = 0.32* 0.14–0.48
The ACL-QOL score should be at least moderately, positively correlated with KOOS Pain subscale scores (r ≥ 0.30)	961	*r* = 0.70* 0.66–0.73	302	*r* = 0.68* 0.63–0.73
The ACL-QOL score should be at least moderately, positively correlated with KOOS Sport/Rec subscale scores (r ≥ 0.30)	955	*r* = 0.74* 0.71–0.77	301	*r* = 0.75* 0.69–0.80
The ACL-QOL score should be at least moderately (r ≥ 0.30), positively correlated with the EQ-5D index score	291	*r* = 0.62* 0.55–0.68	300	*r* = 0.56* 0.46–0.65
Patients who were satisfied with current knee function should report better ACL-QOL scores than those who were not (mean ≥ 10 points)	1103	MD = 33.0* 30.2–35.9	511	MD = 34.7* 30.9–38.5
Patients who returned to pre-injury sport should report better ACL-QOL scores than those who did not (mean ≥ 10 points)	974	MD = 11.8* 9.3–14.3	488	MD = 27.8 23.9–31.7

Results are reported as *r*   Pearson correlation (95% CI); *MD *  mean difference

*Correlation (*r*)/Paired-sample *t*-test (MD) is significant at the 0.01 level (2-tailed); SF-36 = The Optum SF™ Health Surveys SF-36; *PCS*  Physical Component Score, *MCS*   Mental Component Score, *KOOS*   Knee Injury and Osteoarthritis Outcome Score, *EQ-5D*   The EuroQol-5D

### Responsiveness

All except one hypothesis was met representing sufficient responsiveness of the ACL-QOL for use in surgically and non-surgically managed cohorts (Table 6). The one hypothesis that was not met was the correlation between change in IKDC scores and change in the ACL-QOL Social/emotional domain (*r* = 0.27 did not meet the prespecified threshold of *r* > 0.30), reported by non-surgically managed individuals (Table 6).

**Table 6:** Evaluation of responsiveness using a construct approach

	Surgical		Non-surgical	
Hypothesis	*n* = *r/*MD (95% CI)		n = *r/*MD (95% CI)	
Change (3–12 months post ACL injury/surgery) in ACL-RSI should be at least moderately positively correlated (*r* > 0.30) with change in ACL-QOL	62	*r* = 0.71* 0.57–0.82	73	*r* = 0.74* 0.61–0.83
ACL-QOL scores at 12 months post ACL injury/surgery, should be higher (mean ≥ 10 points) than ACL-QOL scores at 3 months post ACL injury/surgery	107	MD = 22.4* 19.7–25.2	113	MD = 13.1** 10.0–16.2
Change (3–12 months post ACL surgery) in KOOS-Pain subscale should be at least moderately positively correlated (*r* > 0.30) with change in ACL-QOL	44	*r* = 0.39* 0.11–0.62	36	*r* = 0.70* 0.49–0.84
ACL-QOL scores at 6 months post ACL surgery should be higher (mean ≥ 10 points) than ACL-QOL scores at 6 weeks post ACL surgery	40	MD = 19.6* 15.5–23.7		
Pre-operative ACL-QOL scores should be lower (mean ≤ 10 points) than ACL-QOL scores 12 months post ACL surgery	51	MD = 30.4* 26.2–34.7		
Change (3–12 months post ACL surgery) in SF-36 MCS should be at least moderately positively correlated (*r* > 0.30) with change in ACL-QOL	39	*r* = 0.49* 0.20–0.70		
Change (3–12 months post ACL surgery) in SF-36 PCS should be at least moderately positively correlated (*r* > 0.30) with change in ACL-QOL	39	*r* = 0.63* 0.39–0.79		
Change (1–3 months post ACL injury) in IKDC should be at least moderately positively correlated (*r* > 0.30) with change in ACL-QOL Lifestyle domain			158	*r* = 0.36* 0.21–0.49
Change (1–3 months post ACL injury) in IKDC should be at least moderately positively correlated (*r* > 0.30) with change in ACL-QOL Social/emotional domain			157	*r* = 0.27* 0.12–0.41
Change (1–12 months post ACL injury) in IKDC should be at least moderately positively correlated (*r* > 0.30) with change in ACL-QOL Lifestyle domain			78	*r* = 0.63* 0.47–0.75
Change (1–12 months post ACL injury) in IKDC should be at least moderately positively correlated (*r* > 0.30) with change in ACL-QOL Social/emotional domain			79	*r* = 0.35* 0.14–0.53

Results are reported as *r* = Pearson correlation (95% CI); MD = Mean difference (95% CI);

*Correlation (*r*)/Paired-sample t-test (MD) is significant at the 0.01 level (2-tailed), *ACLRSI * Anterior Cruciate Ligament Return to Sports After Injury; KOOS; Knee Injury and Osteoarthritis Outcome Score; SF-36 = The Optum SF™ Health Surveys SF-36; *PCS*   Physical Component Score, *MCS*   Mental Component Score, *IKDC*   International Knee Documentation Committee Subjective Knee Form

### Floor and ceiling effects

The proportion of respondents with the lowest possible score on each ACL-QOL domain were 0.1% (*n* = 1, Symptoms/physical), 1.1% (*n* = 17, Work-related), 0.7% (*n* = 12, Recreational/sports) 0.5% (*n* = 8, Lifestyle), and 0.9% (*n* = 16, Social/emotional), indicating a negligible floor effect. The percentage of respondents with the highest possible score on each ACL-QOL domain were 7.1% (*n* = 12, Symptoms/physical), 9.5% (*n* = 153, Work-related), 2.0% (*n* = 35, Recreational/sports) 3.0% (*n* = 52, Lifestyle), and 4.7% (*n* = 81, Social/emotional), indicating a negligible or minor ceiling effect. For the total ACL-QOL score, no participants scored the lowest possible value and 1.1% scored the highest possible value (*n* = 19).

## Discussion

We found that the Swedish version of the ACL-QOL has sufficient internal consistency, construct validity, test–re-test reliability and responsiveness, as well as negligible or minor floor and ceiling effects. Model fit parameters did not meet all cut-offs for good model fit, and further investigations into the source of misfit are needed. Overall, these findings suggest that the Swedish version of the ACL-QOL is a suitable measure to evaluate knee-related QOL in people managed with ACL surgery or rehabilitation alone, at varying timepoints following ACL injury.

The measurement properties of an outcome measure are only specific to the population within which they are tested [21]. To increase the generalisability of our findings, we pooled ACL-QOL data from 13 cohorts ranging from 1 month to 37 years after ACL injury. We also stratified results based on time since ACL injury and management strategy, to evaluate the measurement properties of the Swedish ACL-QOL for use in a variety of patient groups. Internal consistency was sufficient [45] at all follow-up points for use in both surgically and non-surgically managed patients. However, Cronbach’s Alpha exceeded 0.90 for the total ACL-QOL score, as well as the recreational/sports and lifestyle domains, suggesting potential redundancy of items [21]. Our Cronbach’s Alpha results align with the English version of the ACL-QOL evaluated pre-operatively and at 6, 12, and 24 months following ACL reconstruction (Cronbach’s Alpha range 0.93–0.98) [6]. These findings suggest that the ACL-QOL could be shortened to evaluate knee-related QOL with fewer questions, which would reduce the length of time required for completion and the burden on participants.

Notably, our study was the first to evaluate internal consistency within specific ACL-QOL domains. We found sufficient internal consistency for each domain when used with surgically and non-surgically managed patients across all follow-up timepoints. This supports the use of individual ACL-QOL domain scores and suggests that items within each domain are interrelated. We also found that the individual domains of the Swedish version of the ACL-QOL had sufficient test–re-test reliability. Furthermore, assessment of structural validity revealed that the 5-factor model (including each domain score) was the best fitting model for use in surgically and non-surgically managed individuals with ACL injury. Based on these findings we recommend reporting individual domain scores, as well as the total ACL-QOL score, when using this instrument in clinical practice and research settings.

A smaller SEM indicates better absolute reliability and the SEM can also be used to aid in interpretation of findings. We found a SEM of 5.6 points for the total ACL-QOL score, which is slightly lower than the SEM reported for the English version of the ACL-QOL within 2 years of ACLR (SEM: 6.16) [6]. For the ACL-QOL domains, SEM ranged between 7.0 and 10.3. Thus, small degrees of change in ACL-QOL scores not exceeding the SEM, may be explained by reasons other than changes in knee-related QOL [39], and this should be taken into consideration when interpretating change in ACL-QOL scores. Additionally, we found only negligible or minor floor and ceiling effects for the total ACL-QOL score, and domain scores. Unfortunately, we were not able to estimate MIC with confidence, due to the poor correlation between the ACL-QOL and the perceived global rating of change anchor. Since the global rating of change question is a general question, responses to this item may be influenced by aspects other than knee-related quality of life. This has also been identified as a limitation when using other global rating of change scales [36]. For the present study, this might be a reason why the correlation to ACL-QOL was low. Future studies aiming to calculate the MIC for the ACL-QOL, using an anchor-based method, might therefore adapt the global rating of change question for patients to recall the change of the construct being measured, and to recall the specific time period of interest [36].

We had very few missing data in our analyses (≤ 1.5% missing data). Nevertheless, since reasons for missing values were not known, we cannot accurately assess selection bias in this sample. It is possible that the missing values represent difficulty understanding an item or lack of applicability for Swedish respondents, although strategies were employed during translation to reduce the likelihood of this. Considering a sample size of 50 is considered adequate for reliability assessment [37] (we had complete data from 41 participants), our results for this analysis may be subject to a large margin of error. CFI and RMSEA values suggest that the model fit could be improved and that the scoring approaches for the ACL-QOL should be investigated in subsequent studies. Since no study to date has performed a confirmatory or exploratory factor analysis of the English version of the ACL-QOL [6], we were unable to evaluate psychometric closeness with the original version. This is likely a reflection of model fit at the time of instrument development and may be related to item redundancy. Future studies would benefit from evaluating modification indices to identify parameters to improve model fit. Additionally, there may be a need for future studies to test for essential unidimensionality and evaluate measurement invariance between surgical and non-surgical groups, as this was not done in the current study.

It should be noted that during the development of the English version of the ACL-QOL, orthopaedic surgeons were used to establish content validity and two items were removed based on the opinion of orthopaedic surgeons [4]. ACL-injured individuals may perceive issues of relevance to their QOL which may not be clear to orthopaedic surgeons. Although the ACL-QOL was found to contain more items of relevance and importance to ACL-injured individuals compared to other commonly used measures, 22% of items were not important to ACL-injured individuals [5]. Additionally, despite being designed for use in chronically ACL deficient individuals [4], the ACL-QOL is frequently used in patients who are managed with ACL reconstruction. When an instrument is used in a different population than the original population for which it was developed, further evaluation should be performed to determine if all items are relevant for this new population [21]. This suggests that further testing of content validity and potential refinement of the ACL-QOL may be beneficial. Considering the potential redundancy of items, a shorter version of the ACL-QOL where selection of items involves engagement with the target population, could reduce participant burden and ensure only items of relevance are included in the measure.

Additionally, we evaluated the measurement properties of two versions of the Swedish ACL-QOL, a version that is comparable with the English version (results reported in this manuscript), and a modified version with an additional item evaluating the impact on the respondents’ sex life (reported in Online Resource 2). Notably, both versions had strong measurement properties for use in ACL-injured people managed with surgical or non-surgical treatment. Therefore, clinicians and researchers may select the most appropriate version to use for their patient or target population, based on age and other lifestyle factors.

## Conclusion

The Swedish version of the ACL-QOL is an appropriate measure to assess knee-related QOL in people managed with ACL surgery or rehabilitation alone, at a variety of timepoints following ACL injury. We found sufficient, construct validity, test–re-test reliability, and responsiveness, and negligible or minor floor and ceiling effects. However, model fit could be improved and further investigation into suboptimal structural validity is required. Based on these findings, we recommend use of the ACL-QOL to evaluate knee-related QOL in individuals with an ACL injury. We also recommend that ACL-QOL subscale scores are reported in addition to the total score when using this measure in clinical and research settings.

## Supplementary Information

Below is the link to the electronic supplementary material.

Supplementary file1 (PDF 103 kb)

Supplementary file2 (PDF 126 kb)

Supplementary file3 (PDF 97 kb)

Supplementary file4 (PDF 685 kb)
