# Supplementary material for: The Swedish version of the Anterior Cruciate Ligament Quality Of Life measure (ACL-QOL): translation and measurement properties
Source: Qual Life Res. 2022 Oct 13;32(2):593–604. doi: 10.1007/s11136-022-03265-1 (PMC9911474; doi:10.1007/s11136-022-03265-1)
Supplement: Supplementary file 1 — Supplementary file1 (PDF 103 kb) [file 11136_2022_3265_MOESM1_ESM.pdf]

The Swedish version of the Anterior Cruciate Ligament Quality of Life Measure (ACL-QOL): translation and measurement properties

S.R Filbay, H Tigerstrand Grevnerts, S Sonesson, H Hedevik, J Kvist

Online Resource 1. The Swedish Version of the ACLQOL

Bedömning av livskvalitet hos personer med främre korsbandsskada.

Datum: ________________ **Personnummer:**______________________________

Namn: ____

Instruktioner: Detta formulär innehåller frågor om hur ditt skadade knä påverkar din livskvalitet. Besvara varje fråga genom att sätta ett kryss på linjen där du tycker det stämmer överens med hur du upplever situationen.

Symtom och fysiska problem

1. Med tanke på din totala knäfunktion, hur stora besvär har du med att knät ger vika? Observera att denna fråga har två delar. (Om du inte har några besvär, sätt ett kryss i rutan □ och gå vidare till fråga 2.)

1a. Hur mycket viker sig ditt knä?

0 ————————————————————————— 100

Knät viker sig helt — Viss känsla av ostadighet

1b. Hur ofta ger ditt knä vika?

0 ————————————————————————— 100

Knät ger alltid vika — Knät ger aldrig vika

2. Hur mycket smärta eller obehag får du i ditt knä i samband med längre tids aktivitet (mer än 30 min.)?

0 ————————————————————————— 100

Svår smärta — Ingen smärta alls

3. Med tanke på din totala knäfunktion, hur besvärad är du av stelhet och/eller rörelsenedsättning i ditt knä?

0 ———————————————————————— 100
Mycket besvärad — Inte alls besvärad

4. Med tanke på styrkan i dina benmuskler, hur svagt är ditt knä?

0 ———————————————————————— 100
Extremt svagt — Inte alls svagt

Arbetsrelaterade problem

Följande frågor ställs utifrån din arbetssituation. Frågorna behandlar din förmåga att fungera i arbetet och hur ditt knä har påverkat din nuvarande arbetssituation. Om du är studerande eller är hemarbetande ta hänsyn till detta samt till eventuellt deltidsarbete. Bedöm de tre senaste månaderna. Om du är arbetslös på grund av andra orsaker än ditt skadade knä sätt ett kryss i rutan ☐, och gå vidare till fråga 9.

5. Hur stora besvär har du, orsakade av ditt knä, när du gör vändningar eller vridningar i ditt arbete? (Sätt kryss längst till vänster om du inte kan arbeta på grund av ditt knä.)

0 ———————————————————————— 100
Stora problem — Inga problem alls

6. Hur stora besvär har du på grund av ditt knä med att sitta på huk vid arbete? (Sätt kryss längst till vänster om du inte kan arbeta på grund av ditt knä.)

0 ———————————————————————— 100
Stora besvär — Inga besvär alls

7. Hur stort bekymmer är det för dig om du inte kan gå till jobbet några dagar på grund av problem med ditt knä eller på grund av att du skadat ditt knä igen? (Sätt kryss längst till vänster om du inte kan arbeta på grund av ditt skadade knä.)

0 ———————————————————————— 100
Extremt stort bekymmer — Inget bekymmer alls

8. Hur stort bekymmer är det för dig att vara borta från skola eller arbete på grund av behandling av ditt skadade knä?

0 ———————————————————————— 100
Extremt stort bekymmer — Inget bekymmer alls

Deltagande i fysisk fritidsaktivitet, motion eller idrott

Följande frågor handlar om din förmåga att utföra och delta i fysisk fritidsaktivitet, motion eller idrott. Ta hänsyn till de tre senaste månaderna.

9. Hur begränsad är du vid plötslig vändning, vridning eller byte av riktning?

0 ————————————————	100
Totalt begränsad	Inte alls begränsad

10. Hur orolig är du att ditt fritids-, motions- eller idrottsutövande kan resultera i att ditt knä blir sämre?

0 ————————————————	100
Extremt oroad	Inte alls oroad

11. Hur reducerad är din nuvarande fritids-, motions- eller idrottsnivå jämfört med innan du skadade ditt knä?

0 ————————————————	100
Mycket reducerad	Inte alls reducerad

12. Med tanke på fysisk fritidsaktivitet, motion eller idrott som du vill utöva, hur mycket har dina förväntningar på detta förändrats på grund av ditt skadade knä?

0 ————————————————	100
Förväntningarna är betydligt lägre	Förväntningarna är inte alls lägre

13. Måste du vara försiktig vid utövande av fysisk fritidsaktivitet, motion eller idrott? (Om du inte kan utöva fysisk fritidsaktivitet, motion eller idrott på grund av ditt knä, sätt ett kryss längs till vänster på linjen.)

0 ————————————————	100
Är alltid försiktig vid utövande av fysisk fritidsaktivitet/motion/ idrott	Är aldrig försiktig vid utövande av fysisk fritidsaktivitet/motion/ idrott

14. Hur rädd är du att ditt knä ska ge vika under fysisk fritidsaktivitet, motion eller idrott? (Om du inte kan delta i fysisk fritidsaktivitet, motion eller idrott på grund av ditt knä, sätt ett kryss längs till vänster på linjen.)

0 ———————————————————————— 100
Extremt rädd — Inte alls rädd

15. Oroar du dig för yttre förhållanden som t ex vått eller ojämnt underlag, hård bana eller typ av golv i träningslokalen när du utövar fysisk fritidsaktivitet, motion eller idrott? (Om du inte kan delta i fysisk fritidsaktivitet, motion eller idrott på grund av ditt knä, sätt ett kryss längs till vänster på linjen.)

0 ———————————————————————— 100
Extremt oroad — Inte alls oroad

16. Upplever du det frustrerande att behöva tänka på ditt knä vid utövande av fysisk fritidsaktivitet, motion eller idrott?

0 ———————————————————————— 100
Extremt frustrerande — Inte alls frustrerande

17. Hur svårt är det för dig att ”ge allt” vid utövande av fysisk fritidsaktivitet, motion eller idrott? (Om du inte kan delta i fysisk fritidsaktivitet, motion eller idrott på grund av ditt knä, sätt ett kryss längs till vänster på linjen.)

0 ———————————————————————— 100
Extremt svårt — Inte alls svårt

18. Är du rädd för att delta i kontaktidrotter på grund av ditt knä? (Sätt ett kryss i rutan □ om du inte deltar i kontaktidrotter på grund av andra orsaker än din knäskada.)

0 ———————————————————————— 100
Extremt rädd — Inte alls rädd

Följande frågor behandlar de två viktigaste fysiska fritids-, motions- eller idrottsaktiviteter som du utövar eller vill utöva. Skriv dem i rangordning.

1.___________________________

2.___________________________

19. Hur begränsad är du att utöva den fysiska fritidsaktivitet, motion eller idrott du satt som nr 1? (Om du inte kan delta i fysisk fritidsaktivitet, motion eller idrott på grund av ditt knä, sätt ett kryss längs till vänster på linjen.)

0 ———————————————————————— 100
Extremt begränsad — Inte alls begränsad

20. Hur begränsad är du att utöva den fysisk fritidsaktivitet, motion eller idrott du satt som nr 2? (Om du inte kan delta i fysisk fritidsaktivitet, motion eller idrott på grund av ditt knä, sätt ett kryss längs till vänster på linjen.)

0 ———————————————————— 100
Extremt begränsad — Inte alls begränsad

Livssituation

Följande frågor behandlar din livssituation generellt med tanke på ditt knä.

21. Måste du av säkerhetsskäl vidta särskilda åtgärder på grund av ditt knä, t ex när du ska bära små barn eller vid arbete i trädgården?

0 ———————————————————— 100
Måste vidta stora åtgärder — Måste inte vidta några åtgärder

22. Hur mycket har din förmåga att träna och hålla dig i form begränsats av ditt knä?

0 ———————————————————— 100
Totalt begränsats — Inte alls begränsats

23. Hur mycket har din livsnjutning begränsats på grund av ditt knä?

0 ———————————————————— 100
Totalt begränsats — Inte alls begränsats

24. Hur ofta gör sig ditt knäproblem påmint?

0 ———————————————————— 100
Alltid — Aldrig

25. Med tanke på aktiviteter som du utför tillsammans med familj/vänner, hur bekymrad är du över ditt skadade knä?

0 ———————————————————— 100
Extremt bekymrad — Inte alls bekymrad

26. Har du ändrat din livsstil för att undvika aktiviteter som skulle kunna skada ditt knä?

0 ———————————————————— 100
Ändrat totalt — Inte alls ändrat

Socialt och känslomässigt

Följande frågor behandlar din attityd och dina känslor i förhållande till ditt knä.

27. Hur stort bekymmer är det för dig att du inte kan tävla i samma utsträckning nu som innan skadan? (Sätt ett kryss längst till höger om du kan tävla i samma utsträckning nu som innan skadan. Sätt ett kryss i rutan □ om du inte tävlade innan knäskadan.)

0 ——————————————————— 100
Extremt stort bekymmer — Inget bekymmer alls

28. Har du haft svårigheter att hantera din knäskada känslomässigt?

0 ——————————————————— 100
Extremt svårt — Inte alls svårt

29. Hur ofta oroar du dig för ditt knä?

0 ——————————————————— 100
Alltid — Aldrig

30. Hur bekymrad är du över att inte kunna lita på ditt knä? Om du litar på ditt knä sätt ett kryss i rutan □

0 ——————————————————— 100
Mycket bekymrad — Inte alls bekymrad

31*. Hur begränsat är ditt sexualliv på grund av ditt knä?

0 ——————————————————— 100
Totalt begränsat — Inte begränsat alls

32. Hur rädd är du för att skada ditt knä igen?

0 ——————————————————— 100
Extremt rädd — Inte alls rädd

* **This is an additional item that is not in the English version of the ACL-QOL. Both the 32-item and 33-item versions have strong psychometric properties for use in ACL-injured people managed with surgical or non-surgical treatment. Therefore, clinicians**

and researchers may select the most appropriate version to use for their patient or target population, based on age and other lifestyle factors.
