# Supplementary material for: The Swedish version of the Anterior Cruciate Ligament Quality Of Life measure (ACL-QOL): translation and measurement properties
Source: Qual Life Res. 2022 Oct 13;32(2):593–604. doi: 10.1007/s11136-022-03265-1 (PMC9911474; doi:10.1007/s11136-022-03265-1)
Supplement: Supplementary file 2 — Supplementary file2 (PDF 126 kb) [file 11136_2022_3265_MOESM2_ESM.pdf]

The Swedish version of the Anterior Cruciate Ligament Quality of Life Measure (ACL-QOL): translation and measurement properties

S.R Filbay, H Tigerstrand Grevnerts, S Sonesson, H Hedevik, J Kvist

Online Resource 2. Psychometric properties for the Swedish ACL-QOL 33-item Version (including additional item)

Participant characteristics

Table 1. Participant characteristics

	≤ 1.5 year follow-up		2-10 year follow-up		15-25 year follow-up		>30 year follow-up		Total	
	Surgical (n=598)	Non-surgical (n=339)	Surgical (n=370)	Non-surgical (n=121)	Surgical (n=42)	Non-surgical (n=35)	Surgical (n=112)	Non-surgical (n=66)	Surgical (n=1122)	Non-surgical (n=561)
(%) female	384 (64%) ^{0}	173 (51%) ^{2}	197 (53%) ^{0}	56 (46%) ^{0}	12 (29%) ^{0}	12 (34%) ^{0}	29 (26%) ^{0}	19 (29%) ^{0}	622 (55%) ^{0}	260 (47%) ^{2}
Age at follow-up (years)	24 (7) ^{2}	27 (8) ^{5}	27 (8) ^{1}	31 (9) ^{0}	45 (5) ^{0}	43 (4) ^{0}	59 (6) ^{0}	59 (7) ^{0}	29 (13) ^{3}	33 (13) ^{5}
Symptoms/physical domain	81 (16) ^{4}	70 (19) ^{0}	78 (18) ^{4}	76 (20) ^{0}	82 (19) ^{1}	81 (18) ^{1}	78 (20) ^{1}	76 (20) ^{0}	80 (17) ^{10}	72 (20) ^{1}
Work-related domain	69 (23) ^{50}	58 (26) ^{11}	72 (23) ^{24}	70 (25) ^{7}	80 (20) ^{0}	81 (21) ^{3}	74 (23) ^{14}	77 (22) ^{8}	71 (23) ^{88}	64 (26) ^{29}
Recreational/sports domain	56 (25) ^{8}	39 (23) ^{5}	59 (24) ^{2}	50 (25) ^{1}	67 (23) ^{0}	60 (31) ^{1}	59 (28) ^{0}	60 (27) ^{0}	58 (25) ^{10}	45 (26) ^{7}
Lifestyle domain	65 (23) ^{11}	51 (24) ^{6}	69 (23) ^{3}	66 (24) ^{0}	79 (20) ^{0}	73 (22) ^{0}	68 (26) ^{1}	70 (25) ^{1}	67 (24) ^{15}	58 (25) ^{7}
Social/emotional domain	60 (21) ^{11}	52 (22) ^{7}	66 (22) ^{3}	64 (22) ^{1}	84 (17) ^{0}	78 (22) ^{0}	75 (23) ^{0}	77 (21) ^{0}	64 (22) ^{14}	59 (24) ^{8}
ACL-QOL Total Score	64 (20) ^{15}	51 (20) ^{7}	66 (20) ^{5}	62 (21) ^{1}	76 (18) ^{0}	71 (23) ^{0}	69 (23) ^{0}	69 (22) ^{0}	65 (21) ^{20}	57 (22) ^{8}

Descriptives are count (%) or mean (standard deviation); numbers in superscript represent the number of cases with missing data

Reliability

Internal consistency

Table 2. Internal consistency for the 33-item version of the Swedish ACL-QOL

		YEARS SINCE ACL INJURY			
	All patients	≤ 1.5 years	2-10 years	15-25 years	>30 years
Surgical					
Total ACL-QOL	0.971 (n=1057)	0.970 (n=542)	0.970 (n=340)	0.963 (n=41)	0.980 (n=96)
Social/emotional	0.887 (n=1148)	0.868 (n=587)	0.888 (n=367)	0.857 (n=42)	0.915 (n=112)
Non-surgical					
Total ACL-QOL	0.975 (n=528)	0.971 (n=322)	0.972 (n=112)	0.972 (n=30)	0.977 (n=57)
Social/ emotional	0.906 (n=562)	0.888 (n=332)	0.888 (n=120)	0.879 (n=35)	0.901 (n=66)

Data represents the Cronbach's alpha

Test re-test reliability and measurement error

Table 3. Reliability of the 33-item version of the Swedish ACL-QOL administrated 1-4 weeks between test and retest

**ACL-QoL Domain**	**n**	**Mean difference** (95% CI)	**SEM** (95% CI)	**ICC** (95% CI)
ACL-QoL total score	37	-4.0 (-6.5 to -1.4)	5.4	0.93 (0.87-0.96)
Social and emotional	37	-5.3 (-9.6 to -0.9)	9.4	0.83 (0.70-0.91)

Construct validity

Structural validity

Table 4. Fit indices for confirmatory factor analysis model, for the 33-item version of the Swedish ACL-QOL

Model fit indices	CFI	RMSEA (95% CI)	SRMR
Surgical (n=1061)			
Model 1: 1 factor	0.792	0.107 (0.104 to 0.109)	0.063
Model 2A: 2 factor (Symptoms+Work, Sport+Lifestyle+SocEmo)	0.823	0.099 (0.096 to 0.101)	0.057
Model 2B: 2 factor (Symptoms+Work+Sport, Lifestyle+SocEmo)	0.808	0.103 (0.100 to 0.105)	0.063
Model 3A: 3 factor (Symptoms+Work+Sport, Lifestyle, SocEmo)	0.822	0.099 (0.097 to 0.102)	0.062
Model 3B: 3 factor (Symptoms+Work, Sport, Lifestyle+SocEmo)	0.843	0.093 (0.091 to 0.095)	0.056
Model 4A: 4 factor (Symptoms+Work, Sport, Lifestyle, SocEmo)	0.857	0.089 (0.087 to 0.091)	0.055
Model 4B: 4 factor (Symptoms, Work, Sport, Lifestyle+SocEmo)	0.847	0.092 (0.090 to 0.095)	0.054
Model 5: 5 factor (Symptoms, Work, Sport, Lifestyle, SocEmo)	0.860	0.088 (0.086 to 0.091)	0.053
Non-surgical (n=534)			
Model 1: 1 factor	0.830	0.101 (0.097 to 0.104)	0.056
Model 2A: 2 factor (Symptoms+Work, Sport+Lifestyle+SocEmo)	0.859	0.092 (0.089 to 0.095)	0.050
Model 2B: 2 factor (Symptoms+Work+Sport, Lifestyle+SocEmo)	0.846	0.096 (0.093 to 0.099)	0.056
Model 3A: 3 factor (Symptoms+Work+Sport, Lifestyle, SocEmo)	0.851	0.095 (0.091 to 0.098)	0.056
Model 3B: 3 factor (Symptoms+Work, Sport, Lifestyle+SocEmo)	0.883	0.084 (0.080 to 0.087)	0.046
Model 4A: 4 factor (Symptoms+Work, Sport, Lifestyle, SocEmo)	0.889	0.082 (0.078 to 0.085)	0.046
Model 4B: 4 factor (Symptoms, Work, Sport, Lifestyle+SocEmo)	0.888	0.082 (0.079 to 0.086)	0.045
Model 5: 5 factor (Symptoms, Work, Sport, Lifestyle, SocEmo)	0.894	0.080 (0.077 to 0.084)	0.044

CFI = comparative fit index; RMSEA = root mean square error of approximation; SRMR = Standardized Root Mean Square Residual; A CFI close to 0.95 or higher, RMSEA close to 0.06 or lower, and a SRMR close to 0.08 or lower, are representative of good fitting models

Hypothesis testing

Table 5. Hypothesis testing to evaluate construct validity in the 33-item version of the Swedish ACL-QOL

**Hypothesis**	**Surgical (n=1163)**	**Non-surgical (n=570)**
The ACL-QOL score should be at least moderately (r≥ 0.3), positively correlated with the PCS SF-36 score	r=0.70** (0.64 to 0.76)	r=0.71** (0.63 to 0.77)
The ACL-QOL score should be at least moderately (r≥ 0.3), positively correlated with the SF-36 MCS score	r=0.39** (0.30 to 0.49)	r=0.33** (0.15 to 0.49)
The ACL-QOL score should be at least moderately, positively correlated with KOOS Pain subscale scores (r≥0.30)	r=0.70** (0.67 to 0.73)	r=0.69** (0.64 to 0.73)
The ACL-QOL score should be at least moderately, positively correlated with KOOS Sport/Rec subscale scores (r≥0.30)	r=0.74** (0.71 to 0.77)	r=0.76** (0.70 to 0.81)
The ACL-QOL score should be at least moderately (r≥ 0.30), positively correlated with the EQ-5D index score	r=0.63** (0.56 to 0.69)	r=0.57** (0.47 to 0.65)
Patients who were satisfied with current knee function should report better ACL-QOL scores than those who were not (mean ≥10 points)	MD=33	MD=34
Patients who returned to pre-injury sport should report better ACL-QOL scores than those who did not (mean ≥10 points)	MD=11	MD=28

Results are reported as *r*=Pearson correlation (95% CI); MD=Mean difference;
* Correlation (*r*) / Paired-sample t-test (MD) is significant at the 0.01 level (2-tailed);
Confirmation of at least 75% of the predefined hypotheses is considered necessary to represent good construct validity; SF-36=The Optum SF™ Health Surveys SF-36; PCS=Physical Component Score; MCS=Mental Component Score; KOOS=Knee Injury and Osteoarthritis Outcome Score; EQ-5D=The EuroQol-5D

Responsiveness

Table 6. Evaluation of responsiveness of the 33-item version of the Swedish ACL-QOL using a construct approach

**Hypothesis**	**Surgical**	**Non-surgical**
Change (3 to 12 months post ACL surgery) in ACL-RSI score should be at least moderately positively correlated (*r* > 0.30) with change in ACL-QOL score	r = 0.719<br>95% CI: 0.566 to 0.818<br>p < .001<br>n = 62	r = 0.734<br>95% CI: 0.606 to 0.825<br>p < .001<br>n = 73
ACL-QOL scores at 12 months post ACL surgery, should be higher (mean ≥1 points) than ACL-QOL scores at 3 months post ACL surgery	Paired-sample t-test<br>Mean diff = 22.7<br>95% CI: 19.6 to 25.0<br>p < .001<br>n = 107	Paired-sample t-test<br>Mean diff = 13.1<br>95% CI: 10.0 to 16.1<br>p < .001<br>n = 113
Change (3 to 12 months post ACL surgery) in KOOS Pain subscale score should be at least moderately positively correlated (*r* > 0.30) with change in ACL-QoL score	r = 0.401<br>95% CI: 0.118 to 0.624<br>p = .007<br>n = 44	r = 0.713<br>95% CI: 0.502 to 0.844<br>p < .001<br>n = 36
ACL-QOL scores at 6 months post ACL surgery should be higher (mean ≥1 points) than ACL-QOL scores at 6 weeks post ACL surgery	Paired-sample t-test<br>Mean diff = 19.6<br>95% CI: 15.6 to 23.6<br>p < .001<br>n = 40	N/A
Pre-operative ACL-QOL scores should be lower (mean ≥1 points) than ACL-QOL scores 12 months post ACL surgery	Paired-sample t-test<br>Mean diff = 29.9<br>95% CI: 25.7 to 34.1<br>p < .001<br>n = 51	N/A
Change (3 to 12 months post ACL surgery) in the SF-36 Mental Component Score should be at least moderately positively correlated (*r* > 0.30) with change in ACL-QOL score	r = 0.503<br>95% CI: 0.222 to 0.706<br>p = .001<br>n = 39	N/A
Change (3 to 12 months post ACL surgery) in the SF-36 Physical Component Score should be at least moderately positively correlated (*r* > 0.30) with change in ACL-QOL score	r = 0.628<br>95% CI: 0.390 to 0.787<br>p < .001<br>n = 39	N/A
Change (1 to 3 months post ACL injury) in IKDC should be at least moderately positively correlated (*r* > 0.30) with change in ACL-QOL Lifestyle domain		n=158<br>r=0.36*<br>0.21 to 0.49
Change (1 to 3 months post ACL injury) in IKDC should be at least moderately positively correlated (*r* > 0.30) with change in ACL-QOL Social/emotional domain		n=157<br>r=0.32*<br>0.17 to 0.45
Change (1 to 12 months post ACL injury) in IKDC should be at least moderately positively correlated (*r* > 0.30) with change in ACL-QOL Lifestyle domain		n=78<br>r=0.63*<br>0.47 to 0.75

Change (1 to 12 months post ACL injury) in IKDC should be at least moderately positively correlated (*r* > 0.30) with change in ACL-QOL Social/emotional domain	n=79<br>r=0.50*<br>0.32 to 0.65

Interpretability

Floor and ceiling effects

Table 7. Floor and ceiling effects for the 33-item Swedish ACL-QOL

		**Count (%)**
ACL-QOL Total Score	Lowest possible score	0 (0)
	Highest possible score	19 (1.1)
Social / Emotional Domain Score	Lowest possible score	4 (0.2)
	Highest possible score	81 (4.7)
