# Supplementary material for: The Swedish version of the Anterior Cruciate Ligament Quality Of Life measure (ACL-QOL): translation and measurement properties
Source: Qual Life Res. 2022 Oct 13;32(2):593–604. doi: 10.1007/s11136-022-03265-1 (PMC9911474; doi:10.1007/s11136-022-03265-1)
Supplement: Supplementary file 3 — Supplementary file3 (PDF 97 kb) [file 11136_2022_3265_MOESM3_ESM.pdf]

The Swedish version of the Anterior Cruciate Ligament Quality of Life Measure (ACL-QOL): translation and measurement properties

S.R Filbay, H Tigerstrand Grevnerts, S Sonesson, H Hedevik, J Kvist

Online Resource 3. The source of data for psychometric evaluation of the Swedish ACL-QOL

	≤ 1.5-year follow-up		2-10-year follow-up		15-25-year follow-up		>30 year-follow-up		Total	
Cohort	Surgical (n=598)	Non-surgical (n=339)	Surgical (n=370)	Non-surgical (n=121)	Surgical (n=42)	Non-surgical (n=35)	Surgical (n=112)	Non-surgical (n=66)	Surgical (n=1122)	Non-surgical (n=561)
Cross-sectional study[1-3]	31	0	146	0	0	0	0	0	177	0
Prospective cohort study[4,5]	64	48	0	19	0	1	0	0	64	68
Prospective cohort study[6]	120	196	0	0	0	0	0	0	120	196
RCT[7,8]	0	0	2	0	42	34	112	66	156	100
Prospective cohort study[19]	17	88	0	0	0	0	0	0	17	88
Cross-sectional study[20, 21]	0	7	33	7	0	0	0	0	33	14
Cross-sectional study[9]	0	0	0	17	0	0	0	0	0	17
RCT[10]	0	0	21	0	0	0	0	0	21	0
Prospective cohort study[11-16]	147	0	98	0	0	0	0	0	245	0
Prospective cohort study[22]	14	0	21	0	0	0	0	0	35	0
Cross-sectional study[17]	4	0	49	3	0	0	0	0	53	3
Cross-sectional study[4]	201	0	0	0	0	0	0	0	201	0
Cross-sectional study[18]	0	0	0	75	0	0	0	0	0	75

Ethical approvals:

[1, 2, 3, 18]: Regional Ethics Board DNR: 2012/425-32

[4, 5]: Regional Ethical Review Board DNR: 2011 450-31

[6]: Regional Ethical Review Board DNR: 2016/44-31

[7]: Regional Ethical Review Board DNR: 2017/119-31

[8, 10]: Patients identified from the referred studies. Linköping University gave ethical approval for Bachelor theses on the ACL-QoL questionnaire

[9]: Regional Ethical Review Board DNR: 2002/95

[11-17]: Regional Ethical Review Board DNR: 2012/24-31, 2013/75-32, 2017/324-32

[19]: Regional Ethical Review Board DNR: 2012/74-32

[20, 21]: Regional Ethical Review Board DNR: 21-04

[22]: Regional Ethical Review Board DNR 2017/324-32

References:

1. Ardern CL, Österberg A, Sonesson S, Gauffin H, Webster KE, Kvist J. Satisfaction With Knee Function After Primary Anterior Cruciate Ligament Reconstruction Is Associated With Self-Efficacy, Quality of Life, and Returning to the Preinjury Physical Activity. *Arthroscopy.* 2016;32(8):1631-1638.e1633.
2. Ardern CL, Osterberg A, Tagesson S, Gauffin H, Webster KE, Kvist J. The impact of psychological readiness to return to sport and recreational activities after anterior cruciate ligament reconstruction. *Br J Sports Med.* 2014;48(22):1613-1619.
3. Kvist J, Osterberg A, Gauffin H, Tagesson S, Webster K, Ardern C. Translation and measurement properties of the Swedish version of ACL-Return to Sports after Injury questionnaire. *Scand J Med Sci Sports.* 2013;23(5):568-575.
4. Tigerstrand Grevnerts H, Gravare Silbernagel K, Sonesson S, et al. Translation and testing of measurement properties of the Swedish version of the IKDC subjective knee form. *Scand J Med Sci Sports.* 2017;27(5):554-562.
5. Sonesson S, Kvist J, Ardern C, Osterberg A, Silbernagel KG. Psychological factors are important to return to pre-injury sport activity after anterior cruciate ligament reconstruction: expect and motivate to satisfy. *Knee Surg Sports Traumatol Arthrosc.* 2016.
6. Kvist J, Gauffin H, Tigerstrand Grevnerts H, et al. Natural corollaries and recovery after acute ACL injury: the NACOX cohort study protocol. *BMJ Open.* 2018;8(6):e020543.

7. Kvist J, Filbay S, Andersson C, Ardern CL, Gauffin H. Radiographic and Symptomatic Knee Osteoarthritis 32 to 37 Years After Acute Anterior Cruciate Ligament Rupture. *Am J Sports Med.* 2020;48(10):2387-2394.
8. Meunier A, Odensten M, Good L. Long-term results after primary repair or non-surgical treatment of anterior cruciate ligament rupture: a randomized study with a 15-year follow-up. *Scand J Med Sci Sports.* 2007;17(3):230-237.
9. Kvist J. Sagittal plane translation during level walking in poor-functioning and well-functioning patients with anterior cruciate ligament deficiency. *Am J Sports Med.* 2004;32(5):1250-1255.
10. Henriksson M, Rockborn P, Good L. Range of motion training in brace vs. plaster immobilization after anterior cruciate ligament reconstruction: a prospective randomized comparison with a 2-year follow-up. *Scandinavian Journal of Medicine & Science in Sports.* 2002;12(2):73-80.
11. Arundale AJH, Kvist J, Hagglund M, Faltstrom A. Jumping performance based on duration of rehabilitation in female football players after anterior cruciate ligament reconstruction. *Knee Surg Sports Traumatol Arthrosc.* 2018.
12. Arundale AJH, Kvist J, Hagglund M, Faltstrom A. Tuck Jump Score Is Not Related to Hopping Performance or Patient-Reported Outcome Measures in Female Soccer Players. *Int J Sports Phys Ther.* 2020;15(3):395-406.
13. Faltstrom A. One ACL injury is enough! Focus on female football players. *Br J Sports Med.* 2017.
14. Faltstrom A, Hagglund M, Kvist J. Factors associated with playing football after anterior cruciate ligament reconstruction in female football players. *Scand J Med Sci Sports.* 2016;26(11):1343-1352.
15. Faltstrom A, Hagglund M, Kvist J. Functional Performance Among Active Female Soccer Players After Unilateral Primary Anterior Cruciate Ligament Reconstruction Compared With Knee-Healthy Controls. *Am J Sports Med.* 2017;45(2):377-385.
16. Faltstrom A, Kvist J, Gauffin H, Hagglund M. Female Soccer Players With Anterior Cruciate Ligament Reconstruction Have a Higher Risk of New Knee Injuries and Quit Soccer to a Higher Degree Than Knee-Healthy Controls. *Am J Sports Med.* 2018:363546518808006.
17. Faltstrom A, Hagglund M, Kvist J. Patient-reported knee function, quality of life, and activity level after bilateral anterior cruciate ligament injuries. *Am J Sports Med.* 2013;41(12):2805-2813.
18. Sonesson S, Österberg A, Gauffin H, Ardern CL, Kvist J, Hägglund M. Low correlation between functional performance and patient reported outcome measures in individuals with non-surgically treated ACL injury. *Physical Therapy in Sport.* 2021;47:185-192.
19. Olsson I. Incidence of anxiety, depression and health related quality of life in an early phase after an anterior cruciate ligament injury. Master Thesis. Linköping University 2010
20. Sporrstedt K. Translation of Anterior Cruciate Ligament – Quality of Life Questionnaire (ACL-QOL) to Swedish and reliability testing. Master Thesis. Linköping University 2006
21. Sjödahl J. Gait analysis in patients after anterior cruciate ligament reconstruction. Master Thesis. Linköping University, 2005
22. Sabetkar S, Hallberg C. Psychological factor differences in male football players in those that resume versus those that do not resume playing after ACL reconstructive surgery. Bachelor Thesis. Karolinska Institute 2018
