# Supplementary material for: The Swedish version of the Anterior Cruciate Ligament Quality Of Life measure (ACL-QOL): translation and measurement properties
Source: Qual Life Res. 2022 Oct 13;32(2):593–604. doi: 10.1007/s11136-022-03265-1 (PMC9911474; doi:10.1007/s11136-022-03265-1)
Supplement: Supplementary file 4 — Supplementary file4 (PDF 685 kb) [file 11136_2022_3265_MOESM4_ESM.pdf]

The Swedish version of the Anterior Cruciate Ligament Quality of Life Measure (ACL-QOL): translation and measurement properties

S.R Filbay, H Tigerstrand Grevnerts, S Sonesson, H Hedevik, J Kvist

Online Resource 4.

Fit indices for confirmatory factor analysis with standardised factor loadings (surgical n=1163)

Fit indices	Model 1<br>1 factor	Model 2A<br>2 factors	Model 2B<br>2 factors	Model 3A<br>3 factors	Model 3B<br>3 factors	Model 4A<br>4 factors	Model 4B<br>4 factors	Model 5<br>5 factors
CFI*	0.790	0.826	0.806	0.821	0.840	0.856	0.843	0.859
RMSEA*	0.110	0.102	0.106	0.102	0.096	0.091	0.095	0.091
90% CI	0.108–0.112	0.099–0.104	0.103–0.108	0.099–0.104	0.094–0.098	0.089–0.094	0.093–0.098	0.089–0.093
SRMR*	0.063	0.057	0.063	0.061	0.056	0.054	0.055	0.052
Chi-square	6983.6	6014.0	6482.1	6010.2	5406.7	4912.0	5312.4	4818.2
df, p-value	464, <0.001	463, <0.001	463, <0.001	461, <0.001	461, <0.001	458, <0.001	458, <0.001	454, <0.001

Standardized factor loadings

Domain	Item	Model 1	Model 2A	Model 2B	Model 3A	Model 3B	Model 4A	Model 4B	Model 5
Symptoms / Physical	Q1a	0.461	0.586	0.461	0.462	0.587	0.583	0.603	0.598
	Q1b	0.489	0.607	0.488	0.488	0.608	0.605	0.625	0.621
	Q2	0.675	0.800	0.676	0.679	0.800	0.802	0.814	0.816
	Q3	0.696	0.809	0.694	0.697	0.809	0.810	0.816	0.818
	Q4	0.665	0.732	0.671	0.673	0.731	0.732	0.735	0.736
Work	Q5	0.652	0.759	0.656	0.658	0.759	0.760	0.801	0.802
	Q6	0.576	0.666	0.579	0.581	0.665	0.666	0.686	0.687
	Q7	0.427	0.464	0.429	0.429	0.464	0.462	0.539	0.536
	Q8	0.429	0.454	0.425	0.425	0.455	0.454	0.527	0.526
Recreational / Sports	Q9	0.818	0.811	0.835	0.836	0.826	0.826	0.826	0.827
	Q10	0.744	0.743	0.747	0.744	0.741	0.739	0.741	0.739
	Q11	0.780	0.782	0.793	0.796	0.799	0.801	0.799	0.801
	Q12	0.732	0.735	0.739	0.739	0.743	0.743	0.743	0.743
	Q13	0.832	0.838	0.851	0.851	0.866	0.867	0.866	0.867
	Q14	0.804	0.807	0.817	0.814	0.823	0.822	0.823	0.822
	Q15	0.753	0.753	0.756	0.755	0.756	0.755	0.756	0.755
	Q16	0.814	0.821	0.802	0.798	0.808	0.806	0.808	0.806
	Q17	0.864	0.870	0.876	0.877	0.890	0.891	0.890	0.891
	Q18	0.812	0.823	0.821	0.819	0.842	0.841	0.842	0.841
	Q19	0.789	0.794	0.793	0.794	0.802	0.803	0.802	0.802
	Q20	0.691	0.690	0.700	0.703	0.699	0.701	0.699	0.701
Lifestyle	Q21	0.674	0.659	0.667	0.690	0.668	0.696	0.668	0.696
	Q22	0.804	0.801	0.786	0.814	0.786	0.813	0.786	0.813
	Q23	0.794	0.796	0.825	0.827	0.825	0.824	0.825	0.824
	Q24	0.755	0.748	0.776	0.764	0.777	0.768	0.777	0.768
	Q25	0.833	0.830	0.848	0.858	0.848	0.858	0.849	0.858
	Q26	0.813	0.819	0.818	0.832	0.817	0.827	0.817	0.827
Social / Emotional	Q27	0.722	0.729	0.753	0.765	0.753	0.766	0.752	0.766
	Q28	0.567	0.571	0.627	0.686	0.626	0.685	0.626	0.685
	Q29	0.806	0.806	0.847	0.901	0.847	0.899	0.847	0.899
	Q30	0.799	0.801	0.827	0.867	0.826	0.867	0.826	0.867
	Q32	0.703	0.712	0.725	0.780	0.724	0.781	0.724	0.781

CFI = Comparative Fit Index; CI = Confidence Interval; df = degrees of freedom; Q = Question; RMSEA = Root Mean Square Error of Approximation; SRMR = Standardized Root Mean Square Residual

* CFI ≥0.95 or RMSEA <0.06 or SRMR <0.08 represents acceptable structural validity

or

Fit indices for confirmatory factor analysis with standardised factor loadings (Non-surgical n=570)

Fit indices	Model 1<br>1 factor	Model 2A<br>2 factors	Model 2B<br>2 factors	Model 3A<br>3 factors	Model 3B<br>3 factors	Model 4A<br>4 factors	Model 4B<br>4 factors	Model 5<br>5 factors
CFI*	0.838	0.868	0.852	0.858	0.890	0.897	0.895	0.902
RMSEA*	0.100	0.091	0.096	0.095	0.083	0.081	0.081	0.079
90% CI	0.097–0.104	0.087–0.094	0.093–0.099	0.091–0.098	0.080–0.087	0.077–0.084	0.078–0.085	0.076–0.082
SRMR*	0.056	0.047	0.056	0.055	0.045	0.043	0.044	0.042
Chi-square	3131.1	2641.0	2898.4	2808.0	2280.9	2161.2	2191.7	2066.7
df, p-value	464, <0.001	463, <0.001	463, <0.001	461, <0.001	461, <0.001	458, <0.001	458, <0.001	454, <0.001

Standardized factor loadings

Domain	Item	Model 1	Model 2A	Model 2B	Model 3A	Model 3B	Model 4A	Model 4B	Model 5
Symptoms / Physical	Q1a	0.495	0.554	0.495	0.496	0.552	0.550	0.570	0.565
	Q1b	0.506	0.606	0.508	0.509	0.603	0.600	0.623	0.617
	Q2	0.629	0.745	0.625	0.627	0.745	0.751	0.772	0.778
	Q3	0.679	0.798	0.669	0.671	0.801	0.805	0.824	0.826
	Q4	0.660	0.723	0.654	0.656	0.724	0.724	0.745	0.742
Work	Q5	0.696	0.790	0.693	0.694	0.788	0.784	0.829	0.829
	Q6	0.628	0.745	0.621	0.623	0.745	0.746	0.783	0.784
	Q7	0.514	0.597	0.510	0.510	0.597	0.594	0.648	0.648
	Q8	0.502	0.556	0.496	0.496	0.557	0.551	0.600	0.599
Recreational / Sports	Q9	0.770	0.765	0.782	0.782	0.772	0.772	0.772	0.772
	Q10	0.713	0.714	0.713	0.712	0.709	0.709	0.709	0.709
	Q11	0.841	0.845	0.849	0.850	0.854	0.855	0.854	0.855
	Q12	0.679	0.685	0.693	0.693	0.704	0.704	0.704	0.704
	Q13	0.862	0.866	0.878	0.878	0.888	0.888	0.888	0.888
	Q14	0.876	0.880	0.887	0.886	0.894	0.894	0.894	0.894
	Q15	0.809	0.813	0.819	0.818	0.826	0.826	0.826	0.826
	Q16	0.890	0.896	0.894	0.893	0.901	0.901	0.901	0.901
	Q17	0.875	0.881	0.888	0.888	0.898	0.898	0.898	0.898
	Q18	0.815	0.822	0.826	0.825	0.838	0.838	0.838	0.838
	Q19	0.821	0.825	0.825	0.825	0.831	0.831	0.831	0.831
	Q20	0.745	0.747	0.742	0.743	0.741	0.741	0.741	0.741
Lifestyle	Q21	0.688	0.676	0.699	0.715	0.703	0.725	0.703	0.724
	Q22	0.828	0.822	0.820	0.840	0.821	0.840	0.822	0.840
	Q23	0.811	0.809	0.838	0.842	0.838	0.838	0.838	0.839
	Q24	0.834	0.828	0.844	0.845	0.846	0.849	0.846	0.849
	Q25	0.835	0.832	0.856	0.860	0.857	0.859	0.857	0.859
	Q26	0.799	0.802	0.803	0.810	0.801	0.801	0.801	0.800
Social / Emotional	Q27	0.776	0.780	0.790	0.804	0.787	0.805	0.787	0.805
	Q28	0.681	0.682	0.724	0.731	0.722	0.731	0.722	0.731
	Q29	0.845	0.843	0.878	0.898	0.877	0.897	0.877	0.897
	Q30	0.850	0.850	0.862	0.886	0.862	0.885	0.862	0.884
	Q32	0.780	0.787	0.785	0.808	0.781	0.809	0.781	0.810

CFI = Comparative Fit Index; CI = Confidence Interval; df = degrees of freedom; Q = Question; RMSEA = Root Mean Square Error of Approximation; SRMR = Standardized Root Mean Square Residual

* CFI ≥0.95 or RMSEA <0.06 or SRMR <0.08 represents acceptable structural validity
